# Involvement of *S*-nitrosylation of actin in inhibition of neurotransmitter release by nitric oxide

**DOI:** 10.1186/1744-8069-5-58

**Published:** 2009-09-29

**Authors:** Jingshan Lu, Tayo Katano, Emiko Okuda-Ashitaka, Yo Oishi, Yoshihiro Urade, Seiji Ito

**Affiliations:** 1 Department of Medical Chemistry, Kansai Medical University, Moriguchi, Japan; 2 Department of Molecular Behavioral Biology, Osaka Bioscience Institute, Osaka, Japan

## Abstract

**Background:**

The role of the diffusible messenger nitric oxide (NO) in the regulation of pain transmission is still a debate of matter, pro-nociceptive and/or anti-nociceptive. *S*-Nitrosylation, the reversible post-translational modification of selective cysteine residues in proteins, has emerged as an important mechanism by which NO acts as a signaling molecule. The occurrence of *S*-nitrosylation in the spinal cord and its targets that may modulate pain transmission remain unclarified. The "biotin-switch" method and matrix-assisted laser desorption/ionization time-of-flight mass spectrometry were employed for identifying *S*-nitrosylated proteins.

**Results:**

Here we show that actin was a major protein *S*-nitrosylated in the spinal cord by the NO donor, *S*-nitroso-*N*-acetyl-DL-penicillamine (SNAP). Interestingly, actin was *S*-nitrosylated, more in the S2 fraction than in the P2 fraction of the spinal homogenate. Treatment of PC12 cells with SNAP caused rapid *S*-nitrosylation of actin and inhibited dopamine release from the cells. Just like cytochalasin B, which depolymerizes actin, SNAP decreased the amount of filamentous actin cytoskeleton just beneath the membrane. The inhibition of dopamine release was not attenuated by inhibitors of soluble guanylyl cyclase and cGMP-dependent protein kinase.

**Conclusion:**

The present study demonstrates that actin is a major *S*-nitrosylated protein in the spinal cord and suggests that NO directly regulates neurotransmitter release by *S*-nitrosylation in addition to the well-known phosphorylation by cGMP-dependent protein kinase.

## Background

Nitric oxide (NO) is produced from L-arginine by 3 isoforms of NO synthase (NOS), i.e., neuronal NOS (NOS-1), inducible NOS (NOS-2), and endothelial NOS (NOS-3); and it plays important roles in a wide variety of physiological and pathophysiological processes such as neurotransmission, regulation of vascular tone, and mediation of immune responses [1,2]. The major intracellular receptor for NO is a soluble guanylyl cyclase that catalyzes the synthesis of cGMP. This intracellular signaling molecule modulates the activity of many targets in the cells including cGMP-dependent protein kinase (cGK), ion channels, and phosphodiesterases. In the central nervous system, NO is mainly produced by NOS-1 and has been implicated in synaptic plasticity including long-term potentiation in the hippocampus and in pain transmission in the spinal cord [3-5]. Many behavioral studies including ours have demonstrated that NO contributes to the development and maintenance of hyperalgesia and allodynia in models of acute and chronic pain, which are relieved by the blockade of the NO/cGMP/cGK signaling pathway in the spinal cord [6-9]. A rapid release of citrulline, a marker of NO synthesis, is observed in the spinal cord following a subcutaneous injection of formalin and is associated with a biphasic flinching behavior of the injected paw [10]. On the other hand, spinally administered NO donors cause a depression of ongoing impulse activity of dorsal horn neurons [11]; and inhibition of spinal NOS leads to increased neuronal activity in the dorsal horn [12]. Furthermore, agents affecting NO and cGMP levels show no effect [13] or dual effects on nociception depending on their concentrations [14,15]. Thus the involvement of NO in pain is not consistent and is still a matter of debate. Different from many conventional neurotransmitters that are stored in synaptic vesicles and released by exocytosis, the labile, free-radical mediator NO simply diffuses from the nerve terminal into adjacent cells and acts as anterograde and retrograde messengers at nociceptive synapses in the spinal cord [3]. Therefore, the mechanisms through which NO mediates its nociception and pain transmission are not completely understood in the spinal cord [16].

In addition to the NO/cGMP/cGK signaling pathway, *S*-nitrosylation by NO, i.e., the covalent attachment of a -NO group to a cysteine thiol, has emerged as an important feature of NO signaling [17,18]. Through this reversible post-translational modification, NO is able to regulate the function of many target enzymes, ion channels, and structural proteins including cyclooxygenase-2 [19], soluble guanylyl cyclase [20], NMDA receptors [21-23], actin [17,24], and other pathogenic proteins [25,26]. Among methods for studying protein *S*-nitrosylation, the biotin-switch method has rapidly gained popularity because of the ease with which it can detect individual *S*-nitrosylated proteins in biological samples [17,27]; and the use of this method has revealed that *S*-nitrosylation is involved in the physiology and pathophysiology of the central nervous system [18]. Although the NO/cGMP/cGK signaling pathway has been extensively examined in terms of the involvement of NO in nociception and pain transmission, *S*-nitrosylation has not been studied in the spinal cord so far. Here, by using the biotin-switch method, we demonstrate that actin is a major *S*-nitrosylated protein in the spinal cord by the NO donor *S*-nitroso-*N*-acetyl-DL-penicillamine (SNAP) and that interestingly, actin was *S*-nitrosylated, more in the S2 fraction than in the P2 fraction of the spinal homogenate.

## Methods

### Materials

SNAP, nerve growth factor (NGF), 1*H*-[1,2,4]oxadiazolo-[4,3-a]quinoxalin-1-one (ODQ) and KT5823 were obtained from Wako Pure Chemical (Osaka, Japan). Trifluoroacetic acid (TFA), *S*-methyl methanethiosulfonate (MMTS), α-cyano-4-hydroxycinnamic acid (α-CHCA), bovine serum albumin (BSA), imipramine hydrochloride, dopamine, 8-bromoadenosine 3', 5'-cyclic monophosphate (8-Br-cAMP), 8-Br-cGMP, and glibenclamide were purchased from Sigma-Aldrich (St. Louis, MO, USA). Pituitary adenylate cyclase-activating polypeptide (PACAP) and *N*-[6-(biotinamido)hexyl]-3'-(2'-pyridyldithio)propionamide (biotin-HPDP) were supplied by Peptide Institute (Osaka, Japan) and Pierce Chemical (Rockford, IL, USA), respectively. Other chemicals were of reagent grade.

### Preparation of S2 and P2 fractions from spinal cords

Male ddy mice (5 weeks old) were purchased from Shizuoka Laboratory Centre (Hamamatsu, Japan). The mice were housed under conditions of a 12-h light-12-h dark cycle, a constant temperature of 22 ± 2°C, and 60 ± 10% humidity. They received food and water *ad libitum*. All animal experiments were carried out in accordance with the National Institutes of Health guide for the care and use of laboratory animals and were approved by the Animal Experimentation Committee of Kansai Medical University.

Under anesthesia with pentobarbital (50 mg/kg), mouse spinal cords were quickly removed and homogenized twice for 30 s with a Polytron homogenizer containing 10 volumes of HEN buffer consisting of 250 mM HEPES (pH 7.7), 1 mM EDTA, and 0.1 mM neocuproine. The homogenate was centrifuged at 800 × g for 10 min, and the supernatant was recovered and then centrifuged at 10,000 × g for 20 min. After the resulting pellet had been dissolved in 10 volumes of HEN buffer, the resulting supernatant and this dissolved pellet were employed as S2 and P2 fractions, respectively.

### S-Nitrosylation of proteins in vitro and biotin-switch method

*S*-Nitrosylated proteins were detected by the biotin-switch method as described by Jaffrey *et al. *[17]. Briefly, S2 and P2 fractions of the spinal cord were incubated at room temperature without or with various concentrations of SNAP for 1 h in the dark. Then, SNAP was removed from the reaction mixture by cold acetone precipitation; and the pellets were subsequently dissolved in HENS buffer containing 25 mM HEPES, pH 7.7, 0.1 mM EDTA, 0.01 mM neocuproine, and 1% sodium dodecyl sulfate (SDS). The SNAP-treated fractions were blocked with fresh 4 mM MMTS for 20 min at 50°C. After 2 steps of acetone precipitation, the pellets were then resuspended in HENS buffer. For labeling, the samples were subjected to the biotin-switch assay, in which the sample was mixed with 1 mM ascorbic acid and 0.2-0.4 mM biotin-HPDP as final concentrations and kept for 1 h in the dark. Biotinylated proteins were resolved by non-reducing SDS-polyacrylamide gel electrophoresis (PAGE), and transferred to a polyvinylidene difluoride membrane, followed by immunoblotting with peroxidase-conjugated anti-biotin antibody (1:1000; Sigma-Aldrich). To confirm and quantify *S*-nitrosylated actin in the S2 fraction of the spinal cord or cell lysates, after the *S*-nitrosylated proteins had been immunoblotted with anti-biotin antibody, the same membrane was stripped to detect total actin with anti-actin antibody (1:5000; BD Bioscience, San Jose, CA, USA) by using Enhanced Chemiluminescence (GE Healthcare, Piscataway, NJ, USA). The intensity of *S*-nitrosylated actin was quantified by using ImageJ software and normalized by that of total actin.

Furthermore, to detect individual *S*-nitrosylated proteins including actin in S2 fractions of the spinal cord, we removed free biotin-HPDP by using an NAP-5 column (GE Healthcare) after the biotin-switch assay and incubated the eluate overnight at 4°C with 50 μl of a streptavidin-agarose slurry in 2 volumes of a neutralization buffer (20 mM HEPES, pH 7.5, 100 mM NaCl, 1 mM ETDA, and 0.5% Triton X-100). The beads were washed 4 times with 1 ml of the neutralization buffer supplemented with 500 mM NaCl. The adsorbed proteins were eluted with SDS-sample buffer at room temperature for 20 min. The eluted sample was then analyzed by SDS-PAGE, followed by immunoblotting with anti-actin antibody (1:5000).

### Identification of S-nitrosylated proteins by mass spectrometry

Most of the sample eluted from streptavidin-agarose gels was applied to a 10% gel for SDS-PAGE and used for identification of *S*-nitrosylated proteins. After the gel had been stained by the Vorum silver staining protocol [28], proteins of interest were excised from the gel, destained, and in-gel digested as described by Katano *et al*. [29]. The gel piece was dehydrated with acetonitrile and dried by using a Tomy CC-180 vacuum centrifuge concentrator (Tokyo, Japan). After reduction with 10 mM dithiothreitol and alkylation with 55 mM iodoacetamide, proteins in the gel were digested overnight at 37°C with 10 μg/ml trypsin (Promega, Madison, WI, USA) in 25 mM NH_4_HCO_3_. The digested peptides were extracted with 50% acetonitrile in 1% TFA. Peptides were desalted and concentrated by using a Ziptip μC_18 _(Millipore, Billerica, MA, USA) and eluted with the matrix solution (1 mg/ml α-CHCA in 70% acetonitrile and 1% TFA) onto a target plate. Matrix-assisted laser desorption/ionization reflection time-of-flight mass spectrometry (MALDI-TOF MS) was performed by using a Voyager DE-PRO (Applied Biosystems). All spectra were obtained in a positive reflector mode using an accelerating voltage of 20 kV. Database searches were carried out by using the MASCOT search program  and NCBI protein databases. The specified taxonomy was *Mus musculus *(house mouse), and the specified initial mass tolerance was 50 or 70 ppm.

### Cell culture and S-nitrosylation in cells

Pheochromocytoma cell line PC12 cells were maintained in Dulbecco's modified Eagle medium supplemented with 5% fetal calf serum, 10% horse serum, and 50 U/ml penicillin and kept in a humidified environment of 95% air and 5% CO_2 _at 37°C. For the *S*-nitrosylation assay, PC 12 cells were cultured on 6-cm dishes for 2 d; and the medium was replaced with serum-free medium 12 h prior to experiments. After 5-min treatment of the cells with PACAP (10 nM) and/or SNAP (100, 300, 500 μM), the cells were disrupted in HEN buffer by sonication; and the lysate was then subjected to the biotin-switch assay.

### Measurement of dopamine release from PC12 cells

PC12 cells were seeded on 24-well plates. After 2 days in culture, the cells were preincubated for 15 min in 190 μl of HEPES buffer (140 mM NaCl, 5 mM KCl, 2 mM CaCl_2_, 1.2 mM MgCl_2_, 10 mM glucose, and 10 mM HEPES, pH 7.4); and then the appropriate agents (10 μl) were added to the medium. Incubation was carried out 37°C for the desired times in the absence or presence of 10 μM imipramine, an inhibitor of dopamine reuptake. After incubation, the culture medium in each well was harvested; and perchloric acid in HEPES buffer was then added to each well for a final concentration of 3%. Culture media and cell lysates were adjusted to pH 4 by 1 M sodium acetate, and then the samples were centrifuged at 15,000 × g for 5 min. The supernatants of culture media and cell lysates were measured for dopamine released into the medium and cellular dopamine by using an HPLC column equipped with an Eicom electrochemical detector model 700 (Kyoto, Japan). HPLC was performed by using a reversed-phase C18 column (Eicom CA-50DS, 2.1 mm × 150 mm) with a phosphate-buffered mobile phase containing 20% methanol, 50 mg/L EDTA, and 0.5 mg/L sodium 1-octanesulfonate. Cellular dopamine content was around 7.2 ± 0.8 ng/well; and basal and PACAP-evoked release of dopamine into the culture media were 1.5-2 and 15-20% of cellular dopamine, respectively.

### Immunoblot

Cerebellum and dorsal root ganglia (DRG) of mice and PC12 cells were homogenized in 20 mM Tris-HCl (pH 7.4) containing 150 mM NaCl, 4 mM EDTA, 0.5 mM phenylmethylsulfonyl fluoride, 1 μg/ml pepstatin A, 2 μg/ml aprotinin, and 2 μg/ml leupeptin; and the supernatants (100 μg) obtained after centrifugation at 10,000 × g for 30 min were subjected to immunoblotting on a polyvinylidene difluoride membrane. After blocking with 5% (w/v) BSA in TBS-T buffer at room temperature for 1 h, the membrane was incubated overnight at 4°C with rabbit anti-cGKI (type I cGK) antibody (1:200; Santa Cruz Biotech., Santa Cruz, CA, USA) and then at room temperature for 1 h with horseradish peroxidase-conjugated anti-rabbit-IgG (1:20,000; GE Healthcare). The immunoreactivity was detected by using Enhanced Chemiluminescence.

### Fluorescence images for actin in PC12 cells

PC12 cells (3 × 10^4 ^cells/well) were plated on poly-L-lysine-coated glass-bottomed dishes (35 mm) and caused to differentiate with NGF (50 ng/ml) in Dulbecco's modified Eagle medium supplemented with 1% fetal calf serum and 2% horse serum for 4 days. After the cells had been cultured overnight in serum-free medium, they were incubated without or with SNAP (10, 30, 100 μM) or cytochalasin B (10 μM) for 5 min. After fixation in 4% paraformaldehyde in 0.12 M sodium phosphate buffer, pH 7.4 for 10 min, incubation with 0.3% Triton X-100 in phosphate-buffered saline (PBS) for 15 min, 3 washings with PBS, and blocking with 2% normal goat serum and 1% BSA in PBS for 30 min, the PC12 cells were stained for actin. For this procedure, the cells were incubated for 2 h at room temperature with Alexafluora 488-phalloidin (1:500, Invitrogen, Eugeme, OR, USA) alone or with anti-actin monoclonal antibody (1:800), and then for 1 h with anti-mouse IgG-Alexafluora 546 antibody (1:500) in PBS. Digital images were captured by a Zeiss LSM510 laser-scanning confocal microscope (Oberkochen, Germany), and the fluorescence intensity was quantified by using ImageJ. More than 40 cells were quantified at each datum point, and 3 experiments were carried out for each analysis.

### Statistics

Data were presented as the mean ± SD or mean ± SEM. Data for dopamine release and *S*-nitrosylation of actin were analyzed by paired Student's t-test and Mann-Whitney U-test, respectively. Data for F-actin level were analyzed by one-way ANOVA and statistical significance was further examined by Dunnett's test using a Statview software program. P < 0.05 was considered significant.

## Results

### Identification of S-nitrosylated proteins with SNAP in the spinal cord

**Figure 1:**
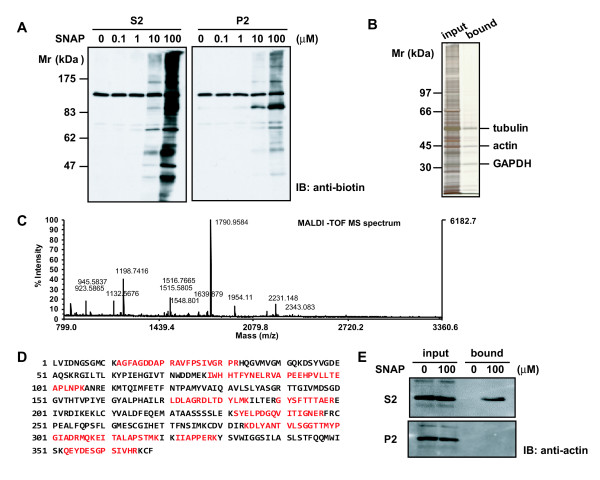
**Identification of protein *S*-nitrosylated by SNAP in the spinal cord as actin**. **A**. S2 and P2 fractions of mouse spinal cords were incubated with the indicated concentrations of SNAP for 1 h and subjected to the biotin-switch assay for *S*-nitrosylation of proteins. Samples (5 μg of each) were resolved by non-reducing SDS-PAGE and immunobloted with anti-biotin antibody. **B**. The S2 fraction was incubated with 100 μM SNAP for 1 h and subjected to the biotin-switch assay. The biotinylated proteins were purified by using a streptavidin-agarose gel, and the bound proteins were eluted by SDS-sample buffer containing 2-mercaptoethanol. Eluates were analyzed by SDS-PAGE, and proteins were visualized by silver staining. Three major bands were identified as β-tubulin, β- and γ-actin, and glyceraldehyde-3-phosphate dehydorogenase (GAPDH). **C**. MALDI-TOF MS spectrum used for identification of actin. Twelve matched mass peaks are indicated. **D**. Amino acid sequence of mouse γ-actin. The parts of the sequence in red correspond to the peptide fragments obtained by tryptic digestion. The amino acid sequence of β-actin completely matches residues 20-351 of γ-actin except that P at the 31st residue of γ-actin was replaced by S at the 12th residue of β-actin. **E**. *S*-Nitrosylation of actin by SNAP in the S2 fraction, but not in the P2 fraction, of the spinal cord. S2 and P2 fractions of spinal cords were incubated with 100 μM SNAP for 1 h and subjected to the biotin-switch assay. After removing free biotin-HPDP, *S*-nitrosylated proteins were purified on streptavidin-agarose gels. Eluates were immunoblotted with anti-actin antibody as described in "Methods."

To identify *S*-nitrosylated proteins in the spinal cord, we separated homogenates of the spinal cord into supernatant (S2) and pellet (P2) fractions after a 20-min centrifugation at 10,000 × g. They were then incubated for 1 h with various concentrations of SNAP, an NO donor, and subjected to the biotin-switch assay for *S*-nitrosylation. As shown in Figure 1A, *S*-nitrosylated proteins were detected with anti-biotin antibody in both S2 and P2 fractions in a concentration-dependent manner; and the extent of *S*-nitrosylation in the S2 fraction was much higher than that in the P2 fraction at 10 and 100 μM SNAP. Next, biotinylated proteins in the S2 fraction treated with 100 μM SNAP were purified on a streptavidin-agarose gel and selectively eluted by using SDS-sample buffer containing 2-mercaptoethanol. Three major bands appeared after SDS-PAGE (Figure 1B) and were in-gel digested and then subjected to MALDI-TOF MS. The mass data were processed to assign candidate peptides in the NCBI database by using the MASCOT search program. These three major bands were identified as β-tubulin, β- and γ-actin, and glyceraldehyde-3-phosphate dehydrogenase (Table 1). Figure 1C shows the mass spectrum corresponding to tryptic digests of actin. The sequence coverage of γ-actin was 39%, and the probability-based MOWSE score was 130 with 12 matched peptides. Since the amino acid sequences are highly conserved between β- and γ-actin, these tryptic peptides did not discriminate them except for the N-terminal portion (Figure 1D).

**Table 1:** Proteins identified by MALDI-TOF MS

**No**	**Identified protein**	**Mr (Da)**	**% cover**	**Pept matched**	**Mowse score**	**NCBI GI number**
1	β-tubulin	50,281	27	12	96	4507729
2	γ-actin	41,340	39	12	130	809561
	β-actin	39,451	39	12	131	49868
3	GAPDH^a)^	31,120	35	7	82	1.5E+08

^a)^GAPDH = glyceraldehyde-3-phosphate dehydorogenase.

To confirm that *S*-nitrosylated protein identified by MALDI-TOF MS was indeed actin, we immunoblotted the eluate from the streptavidin-agarose gel with anti-actin antibody. *S*-nitrosylated actin was detected in the eluate of the S2 fraction treated with 100 μM SNAP (Figure 1E, *upper panel*). Interestingly, however, it was not found in the P2 fraction (Figure 1E, *lower panel*), suggesting that *S*-nitrosylation was affected by the state of actin.

### Effect of SNAP on dopamine release from PC12 cells

**Figure 2:**
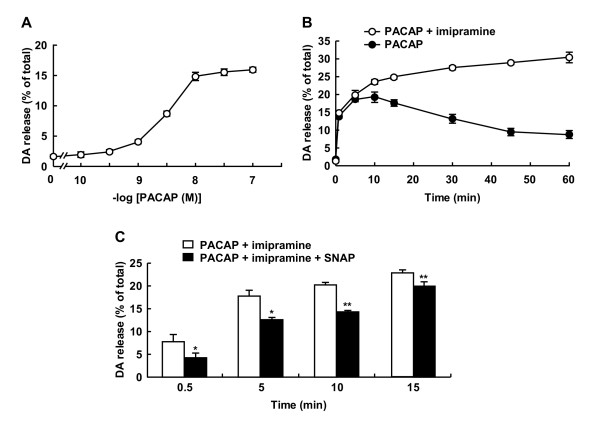
**Inhibition of PACAP-stimulated dopamine release from PC 12 cells by SNAP**. **A**. Concentration dependency of PACAP for dopamine release from PC12 cells. PC12 cells (4 × 10^5 ^cells/well) cultured on 24-well dishes were stimulated with various concentrations of PACAP for 5 min. **B**. Time courses of dopamine release from PC12 cells by PACAP. PC12 cells were stimulated for the indicated times with 10 nM PACAP in the absence (closed circles) or presence (open circles) of 10 μM imipramine, an inhibitor of catecholamine reuptake. **C**. Inhibition of PACAP-stimulated dopamine release by SNAP. PC12 cells were stimulated with 10 nM PACAP without (open columns) or with (closed columns) 100 μM SNAP in the presence of 10 μM imipramine for the indicated times. Dopamine released into the medium and cellular dopamine were measured by HPLC as described in "Methods." Dopamine (DA) release (mean ± SD, n = 3) was expressed as a percentage of total dopamine (7.2 ± 0.8 ng/well) in PC12 cells. *P < 0.05; **P < 0.01 vs. without SNAP.

Acting as a cell-permeable intercellular messenger, NO is known to affect neurotransmitter release. PC12 cells are a rat pheochromocytoma cell line that endogenously produces dopamine and shares similar mechanisms of exocytosis with neurons [30]. Dopamine is the predominant catecholamine in PC12 cells [31], and PACAP is known to stimulate the release of dopamine from PC12 cells. To study whether SNAP affected the release of dopamine from PC12 cells, we first examined the release elicited by PACAP by using an HPLC column equipped with an electrochemical detector. The basal release of dopamine into the medium represented about 1.5-2% of the total cellular dopamine in PC12 cells. Exposure to PACAP for 5 min stimulated the release of dopamine in a concentration-dependent manner, with an EC_50 _value of 2.42 nM; and the release started to increase at 1 nM and reached the maximum at 10 nM, at which concentration 15-20% of the total cellular dopamine was released (Figure 2A). When PC12 cells were treated with 10 nM PACAP, the release reached the maximum at 5-10 min and gradually decreased for 60 min (Figure 2B). In the presence of 10 μM imipramine, an inhibitor of dopamine reuptake, 10 nM PACAP stimulated the release for 60 min in a time-dependent manner, suggesting that dopamine released into the medium was reuptaken for a longer incubation. When the effect of SNAP on dopamine release stimulated by 10 nM PACAP was examined in the presence of imipramine, 100 μM SNAP inhibited PACAP-induced dopamine release from PC12 cells by 47.6, 30.4, 30.5, and 13.2% at 0.5, 5, 10, and 15 min after stimulation, respectively (Figure 2C). Since the inhibitory effect decreased with time, the effect of 100 μM SNAP on PACAP-evoked dopamine release was examined at 5 min in the absence of imipramine in subsequent experiments.

### No involvement of NO/cGMP/cGK signaling pathway in the inhibitory effect of SNAP on dopamine release

**Figure 3:**
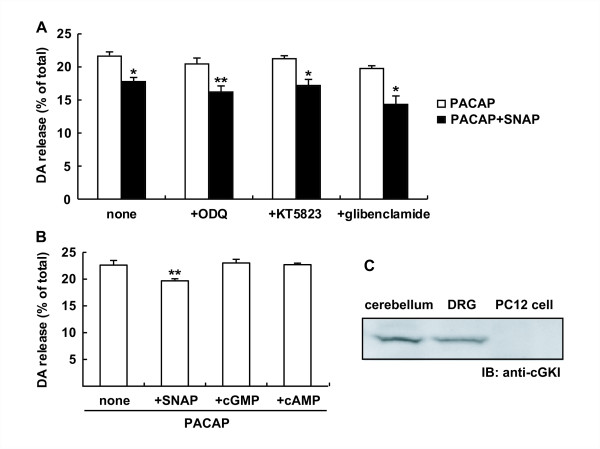
**No mediation of the cGMP/cGK pathway in inhibition of dopamine release by SNAP**. **A**. Effect of inhibitors of the NO/cGMP/cGK pathway on attenuation of dopamine release by SNAP. PC12 cells (4 × 10^5 ^cells/well) were stimulated for 5 min with 10 nM PACAP without (open columns) or with (closed columns) 100 μM SNAP in the presence of 300 nM ODQ, 1 μM KT5823 or 1 μM glibenclamide. **B**. Effect of cGMP and cAMP on dopamine release stimulated by PACAP. PC12 cells were stimulated for 5 min with 10 nM PACAP in the presence of 100 μM SNAP, 100 μM 8-Br-cGMP or 100 μM 8-Br-cAMP. Dopamine was measured by HPLC, and dopamine (DA) released into the medium (mean ± SD, n = 3) was expressed as a percentage of total dopamine in PC12 cells as described in the legend for **Figure 2**. *P < 0.05; **P < 0.01 vs. PACAP alone. **C**. Expression of cGKI in tissues and PC12 cells. Aliquots (100 μg/lane) of supernatants after centrifugation of homogenates of tissues and PC12 cells at 10,000 × g for 30 min were applied to 7.5% SDS-PAGE and immunoblotted with anti-cGKI antibody.

In addition to recent findings of *S*-nitrosylation of cysteine residues of specific proteins, NO has long been known to bind to soluble guanylyl cyclase, leading to cGMP production and cGK activation. To clarify whether the suppressive effect of SNAP on the dopamine release was mediated by the NO/cGMP/cGK signaling pathway, we examined whether the suppressive effect by SNAP could be attenuated by ODQ, a guanylyl cyclase blocker; KT5823, an inhibitor of cGK; or glibenclamide, an ATP-sensitive K^+ ^channel blocker [32]. When the suppressive effect of SNAP was assessed at 5 min in the absence of imipramine, ODQ (300 nM), KT5823 (1 μM) or glibenclamide (1 μM) affected neither the enhancement of dopamine release by PACAP nor the suppression of the release by SNAP (Figure 3A). To further examine whether cGMP could mimic the inhibitory effect by SNAP, we applied 100 μM 8-Br-cGMP and 8-Br-cAMP, permeable analogs of cGMP and cAMP, respectively, to the cells instead of SNAP. Different from SNAP, 8-Br-cGMP and 8-Br-cAMP did not affect the dopamine release evoked by PACAP (Figure 3B), demonstrating that the attenuation of PACAP-enhanced dopamine release by SNAP was independent of the NO/cGMP/cGK signaling pathway in PC12 cells.

To further clarify that the suppressive effect by SNAP was not mediated by cGK, we examined the expression of cGK in PC12 cells by immunoblotting with anti-cGKI antibody. While it was detected in lysates of the cerebellum and DRG, cGKI was not detected in PC12 cells (Figure 3C).

### Inhibition of formation of filamentous actin (F-actin) by SNAP

**Figure 4:**
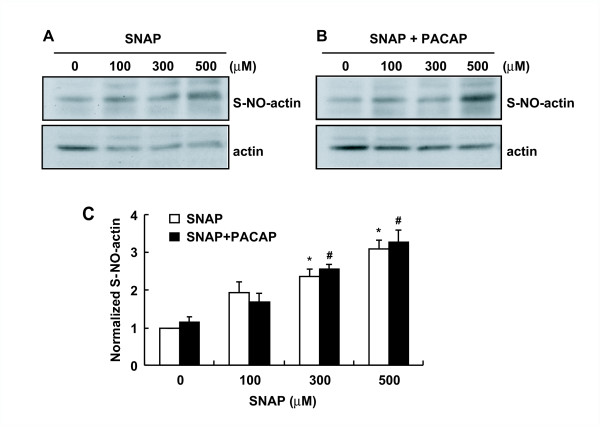
***S*-Nitrosylation of actin by SNAP in PC12 cells**. **A, B**. *S*-Nitrosylation of actin in PC12 cells by SNAP in the absence (**A**) and presence (**B**) of PACAP. PC12 cells (7 × 10^5 ^cells/dish) cultured in 6-cm dishes were exposed to 100, 300 or 500 μM SNAP with 10 nM PACAP for 5 min. The cell lysates in HEN buffer were subjected to the biotin-switch assay and analyzed by SDS-PAGE as described in "Methods." **C**. Quantification of *S*-nitrosylated actin in PC12 cells. Intensity of bands was quantified by ImageJ, and the extent of *S*-nitrosylated actin was normalized to total actin. *S*-Nitrosylated actin by SNAP in the absence (**A**, open columns) and presence (**B**, closed columns) of PACAP is expressed as a ratio (mean ± SEM, n = 3) to that at 0 μM SNAP. *P < 0.05 vs.0 μM SNAP without PACAP; ^#^P < 0.05 vs.0 μM SNAP with PACAP.

To clarify the involvement of *S*-nitrosylation of actin in dopamine release, we next examined whether actin was *S*-nitrosylated with SNAP in PC12 cells. In PC12 cells, actin was *S*-nitrosylated by SNAP in a similar manner, regardless of the presence of 10 nM PACAP (Figure 4A and 4B). When the band intensity of *S*-nitrosylated actin was normalized to that of total actin and the ratio without SNAP treatment was taken as 1, *S*-nitrosylation of actin in PC12 cells rapidly increased 1.93 to 3.11-fold and 1.69 to 3.29-fold during a 5-min incubation in a concentration-dependent manner from 100 to 500 μM SNAP in the absence and presence of PACAP, respectively (Figure 4C). It was hard to detect the increase in *S*-nitrosylated actin above the control at less than 100 μM SNAP by the biotin-switch assay (data not shown).

**Figure 5:**
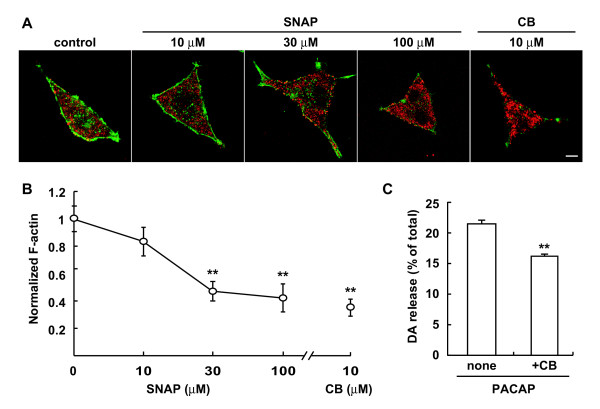
**Inhibition of dopamine release by SNAP through F-actin breakdown**. A. Representative fluorescence images of F-actin distribution in PC12 cells. After PC12 cells (3 × 10^4 ^cells/well) had been treated for 5 d with NGF (50 ng/ml), they were incubated for 5 min with various concentrations of SNAP or 10 μM cytochalasin B (CB). After fixation with 4% paraformaldehyde, the cells were labeled with Alexafluora 488-phalloidin (green) for F-actin and anti-actin monoclonal antibody/Alexafluora 546-anti-mouse IgG (red) for total actin. Bar = 5 m. **B**. Effect of SNAP and cytochalasin B on F-actin formation. Fluorescence intensity of more than 40 cells/slide was quantified by ImageJ as described in "Methods." Data are the mean ± SEM of 3 independent experiments. *P < 0.05, **P < 0.01 vs. 0 M SNAP. **C**. Effect of cytochalasin B on dopamine release stimulated by PACAP. PC12 cells were stimulated for 5 min with 10 nM PACAP without or with 10 M CB, and dopamine release was measured as described in the legend for **Figure 2**. **P < 0.01 vs. without CB.

*S*-Nitrosylation of actin by SNAP was detected in the S2 fraction, but not in the P2 fraction of the spinal cord (Figure 1E). To get more insight into the relationship between *S*-nitrosylation of actin and attenuation of dopamine release by SNAP, we examined the alteration of F-actin in NGF-differentiated PC12 cells. After NGF-differentiated PC12 cells had been treated for 5 min with SNAP (10, 30, 100 μM) or cytochalasin B, a drug that prevents the addition of monomers to F-actin [33], the cells were double-labeled with phalloidin for F-actin and anti-actin antibody for total actin. As shown in Figure 5A, whereas actin (red) was broadly observed in the cell, F-actin (green) was mainly observed beneath the cell membrane and in neurites. The distribution and fluorescence intensity of total actin did not change significantly after the addition of SNAP. When normalized to that of total actin, the fluorescence intensity of F-actin (>40 cells/measurement, n = 3) was rapidly reduced to 81.2, 49.4 and 41.6% by 10, 30, and 100 μM SNAP in a concentration-dependent manner (Figure 5B). Similar results were obtained with the cells treated with cytochalasin B, and the extent of the decrease in F-actin (38.4%) caused by cytochalasin B was similar to that by 100 μM SNAP.

Conversely, we examined whether cytochalasin B would attenuate dopamine release triggered by PACAP. As shown in Figure 5C, cytochalasin B attenuated the dopamine release similarly as 100 μM SNAP, suggesting that SNAP reduced the amount of dopamine release from PC12 cells by breakdown of the F-actin cytoskeleton just beneath the plasma membrane.

## Discussion

Many studies including ours have demonstrated that activation of the NMDA subtype of glutamate receptors and subsequent NO production are a fundamental event in neurotransmission and synaptic plasticity in pain transmission in the spinal cord [3-5]. The classical NO signaling pathway is mediated by the generation of cGMP to regulate cGK. In the present study, by use of the biotin-switch method we first demonstrated that proteins in the homogenate of the mouse spinal cord were *S*-nitrosylated by the NO donor SNAP in a concentration-dependent manner. Interestingly, the extent of *S*-nitrosylation was more prominent in the S2 fraction than in the P2 fraction (Figure 1A). The biotinylated proteins in the S2 fraction were purified by using a streptavidin-agarose gel (Figure 1B); and β- and γ-actin, β-tubulin, and glyceraldehyde-3-phosphate dehydrogenase were identified by MALDI-TOF MS (Figure 1C and 4D, Table 1). These proteins were earlier shown to be *S*-nitrosylated in brain lysates [17]. There are several reports showing the *S*-nitrosylation of actin *in vitro *and *in vivo *and nitrosylated cysteine moieties in the carboxyl terminal area of actin, a region that is important for actin polymerization and for binding several proteins that modify behavior of the molecule [24,34]. One of these sites is identified as Cys^374^, and the nitrosylated G-actin is found to polymerize less efficiently [34]. A number of experiments involving deletions or mutations of actin at the C-terminus have demonstrated the importance of this region for proper F-actin polymerization and stability. For example, mutating Cys^374 ^to Ser in chicken β-cytoplasmic actin increases the critical concentration for polymerization by more than 5-fold [35]. Also, removal of either 2 or 3 of the C-terminal residues of actin results in actin filaments with increased fragility and flexibility [36,37]. In the present study we demonstrated by phalloidin binding that the content of F-actin was rapidly reduced by SNAP in a dose-dependent manner as well as by cytochalasin B, an agent that depolymerizes actin, but that the content of total actin was not changed (Figure 5A and 5B).

### Dopamine release and S-nitrosylation of actin

PC12 cells are a rat pheochromocytoma cell line that synthesizes dopamine endogenously and are a commonly employed cellular model for investigating the neurotrophic effects of PACAP [38]. PC12 cells share a similar mechanism of exocytosis with neurons [30], and the mechanisms underlying the release of dopamine from PC12 cells, including membrane depolarization and increase in intracellular Ca^2+^, have been extensively investigated through reconstitution of PAC1 receptors for PACAP in PC12 cells [39]. As shown in this study, PACAP stimulated dopamine release from PC12 cells in a concentration-dependent manner with an EC_50 _of 2.42 nM (Figure 2A). Dopamine was rapidly released within 1 min after stimulation with PACAP, and reuptake of it into the cells started by 10 min (Figure 2B). Although SNAP attenuated the evoked release at any time from 0.5 to 15 min, the extent of the inhibitory effect by SNAP was reduced during a longer incubation in the presence of imipramine (Figure 2C). Therefore, to simplify the elucidation of the action mechanisms of SNAP, we examined the effect of SNAP on the release for the initial 5 min. NO has long been considered to act largely through cGMP formed by activation of soluble guanylyl cyclase and subsequent cGK activation in the nervous system [5]. It was previously shown that an ATP-sensitive K^+ ^channel is a target of cGK and that glibenclamide directly blocks both acute and persistent hypernociception via opening of an ATP-sensitive K^+ ^channel [32]. The inhibition by SNAP was not attenuated by ODQ, a soluble guanylyl cyclase inhibitor; KT5823, a cGK inhibitor; or glibenclamide, an ATP-sensitive K^+ ^channel blocker (Figure 3A). Conversely, neither 8-Br-cGMP nor 8-Br-cAMP itself affected the basal release (data not shown) or PACAP-enhanced dopamine release (Figure 3B). Consistent with the distribution of cGKIα in the cerebellum and DRG [40], it was detected in the homogenate of the cerebellum and DRG, but not in PC12 cells (Figure 3C). These results demonstrate that the inhibitory effect by SNAP was not mediated by the NO/cGMP/cGK pathway.

### Actin rearrangement and dopamine release

At the nerve terminal, the majority of synaptic vesicles are bound within a layer of F-actin beneath the plasma membrane [41,42]. Upon neuronal stimulation, breakdown of the actin cytoskeleton is required for vesicle movement to the plasma membrane and subsequent neurotransmitter release. Thus, the classical view of actin rearrangements in most secretory cells has been based on the actin-physical-barrier model, whereby local disassembly of the cortical actin network permits secretory granules to gain access to exocytotic sites at the plasma membrane. However, this model has been questioned because actin polymerization may also play an important active role in the final stages of exocytosis [43]. An important mechanism underlying exocytosis is the assembly of distinct pools of synaptic vesicles at release sites, where two pools of synaptic vesicles have been identified, the readily releasable pool and the reserve pool. The readily releasable pool, constituting only a small fraction of the total vesicles, represents those vesicles released during the fast phase of exocytosis [44]. These vesicles are docked at the plasma membrane and released rapidly upon cell stimulation. SNAP also inhibited the KCl-induced domapine release (Lu J, unpublished observation) and cytochalasin B also inhibited the dopamine release evoked by PACAP (Figure 5C). Taken together with our findings that the extent of *S*-nitrosylation was higher in the S2 fraction than in the P2 fraction (Figure 1A) and that the inhibitory effect of SNAP on dopamine release was greater during the early phase (Figure 2C), the present study suggests that the reduction in F-actin by *S*-nitrosylation resulted in inhibition of dopamine release, possibly from the readily releasable pool.

### Two mechanisms of NO action in the spinal cord

We and others have demonstrated that NO contributes to the development and maintenance of hyperalgesia and allodynia in models of acute and chronic pain, which are prevented by blockade of the NO/cGMP/cGK signaling pathway in the spinal cord [6-9]. cGKIα is expressed in small- or medium-diameter neurons of the lumbar DRG, the class of neurons involved in nociceptive processing and in central nerve terminals of laminas I and II in the spinal cord, but is not present in spinal neurons and white matter tracks [40,45]. By contrast, NOS-1 is present in only 1-2% of lumbar DRG neurons; but NOS-1-containing fibers and small interneurons are present in all layers of the spinal cord, especially in lamina II. Therefore, NO has been supposed to act as a retrograde messenger [3,40], i.e., to diffuse back to the presynaptic terminals of primary afferent fibers where it stimulates soluble guanylyl cyclase resulting in the formation of cGMP. It may, in turn, activate cGK, resulting in further glutamate release and NOS-1 activation. We previously demonstrated that NO serves as a retrograde messenger in the spinal cord and stimulates glutamate release from primary afferent terminals through the NO/cGMP/cGK pathway by the use of NO donors such as NOR3 and SNAP [46,47].

In the present study, we first demonstrated that actin in the S2 fraction of the spinal cord was *S*-nitrosylated by exogenous NO; and our data suggest that *S*-nitrosylation attenuated dopamine release by decrease in F-actin content in PC12 cells. It could be deduced that glutamate release can be affected by NO-induced *S*-nitrosylation of actin in the spinal cord. In this connection, the NO donor sodium nitroprusside inhibited ongoing impulse activity in 49% of all spinal neurons in the laminas I and II and activated only 28% of them [11]. Although the biphasic effect of NO on neuronal activity was vaguely considered to be the difference in experimental conditions under which neuronal background activity was examined, it may be simply interpreted that NO affected glutamate release in both inhibitory and stimulatory manners by the balance of *S*-nitrosylation and cGMP/cGK pathway.

## Conclusion

Unlike the other second messengers, NO has the potential to induce opposing effects and previous studies suggest that the NO/cGMP signaling cascade showed both pro- and anti-nociceptive effects [16]. Although which types of cells in the spinal cord are *S*-nitrosylated remains to be clarified, the present study suggests that 2 signaling pathways of NO action exist and that the contribution of these 2 pathways is determined to a considerable extent by the presence of cGK in cells. Two signaling pathways may explain the discrepancy in pain behaviors and electrophysiological responses to NO donors in the spinal cord.

## Abbreviations

biotin-HPDP: N-[6-(biotinamido)hexyl]-3'-(2'-pyridyldithio)-propionamide; 8-Br-cAMP: 8-bromoadenosine 3', 5'-cyclic monophosphate; BSA: bovine serum albumin; a-CHCA: a-cyano-4-hydroxycinnamic acid; cGK: cGMP-dependent protein kinase; DRG: dorsal root ganglia; F-actin: filamentous actin; MALDI-TOF MS: matrix-assisted laser desorption/ionization reflection time-of-flight mass spectrometry; MMTS: S-methyl methanethiosulfonate; NGF: nerve growth factor; NO: nitric oxide; NOS: NO synthase; ODQ: 1H-[1,2,4]oxadiazolo-[4,3-a]quinoxalin-1-one; PACAP: pituitary adenylate cyclase-activating polypeptide; PAGE: polyacrylamide gel electrophoresis; PBS: phosphate-buffered saline; SDS: sodium dodecyl sulfate; SNAP: S-nitroso-N-acetyl-DL-penicillamine; TFA: trifluoroacetic acid.

## Competing interests

The authors declare that they have no competing interests.

## Authors' contributions

JL was involved in data acquisition of *S*-nitrosylation, identification of actin by MALDI-TOF MS, and cell experiments, TK and EO were involved in supervision of JL in all experiments. YO and YU participated experiments of dopamine release. SI is a corresponding author and participated in the design of experiments and manuscript preparation. All authors read and approved the final manuscript.
